# Differences in the Functional Traits of *Populus pruinosa* Leaves in Different Developmental Stages

**DOI:** 10.3390/plants12122262

**Published:** 2023-06-09

**Authors:** Juntuan Zhai, Xiao Zhang, Zhijun Li, Xiaoli Han, Shanhe Zhang

**Affiliations:** Key Laboratory of Protection and Utilization of Biological Resources in Tarim Basin of Xinjiang Production & Construction Corps, College of Life Science and Technology, Tarim University, Alar 843300, China; zhaijuntuan2022@163.com (J.Z.); tlmdxzx@163.com (X.Z.); lilyan0509@163.com (X.H.); zhanghh1127@163.com (S.Z.)

**Keywords:** *Populus pruinosa*, leaf, functional traits, developmental stage, canopy height

## Abstract

*Populus pruinosa* Schrenk has the biological characteristics of heteromorphic leaves and is a pioneer species for wind prevention and sand fixation. The functions of heteromorphic leaves at different developmental stages and canopy heights of *P. pruinosa* are unclear. To clarify how developmental stages and canopy height affect the functional characteristics of leaves, this study evaluated the morphological anatomical structures and the physiological indicators of leaves at 2, 4, 6, 8, 10, and 12 m. The relationships of functional traits to the developmental stages and canopy heights of leaves were also analyzed. The results showed that blade length (BL), blade width (BW), leaf area (LA), leaf dry weight (LDW), leaf thickness (LT), palisade tissue thickness (PT), net photosynthetic rate (Pn), stomatal conductance (Gs), proline (Pro), and malondialdehyde (MDA) content increased with progressing developmental stages. BL, BW, LA, leaf dry weight, LT, PT, Pn, Gs, Pro, and the contents of MDA, indoleacetic acid, and zeatin riboside had significant positive correlations with canopy heights of leaves and their developmental stages. The morphological structures and physiological characteristics of *P. pruinosa* leaves showed more evident xeric structural characteristics and higher photosynthetic capacity with increasing canopy height and progressive developmental stages. Resource utilization efficiency and the defense ability against environmental stresses were improved through mutual regulation of each functional trait.

## 1. Introduction

With the frequent occurrence of global climate change and extreme climate change events, it is particularly important to cultivate plants with wide adaptability to cope with the constantly changing environment using effective methods [1]. Functional traits are attributes of plants, including phenotype, anatomical structure, physiological function, and other traits, that have significant impacts on plant growth and reproduction [2,3,4]. If the change range of plant functional traits is greater, then the plant will be more able to adapt to the complex and changeable environment [5,6], providing more choices of functional traits to study.

Variations in the functional traits of the same species can increase the competitiveness of the species [7]; such variations show differences at different ages to maximize the effective use of limited resources and avoid intraspecific competition [8,9]. The leaf is the organ most easily affected by environmental changes and can best reflect the functions of plants and their adaptability to the environment [5,10,11,12]. As they are the main carbon source of plants, changes in functional traits of leaves play an irreplaceable role in adapting to environmental stress [13,14,15,16]. Regardless of gymnosperms or angiosperms [17,18,19,20], specific leaf weight increases with increasing tree age, which may be due to the vulnerability of the leaves of older trees to water stress [21,22,23]. *Eucalyptus regnans* F. Muell. leaves become more polymorphic with increasing tree age and height; *Sabina vulgaris* Ant. has two leaf forms, scaly leaf and needle leaf, which are different in morphology and physiological characteristics, and the scaly leaf has higher drought resistance than the needle leaf [24]. Significant differences in leaf morphology, structure, and physiological functions of *Populus euphratica* Olivier occur at different developmental stages, and tree height gradients vary at the same developmental stage; these differences are related to the individual developmental stage and the crown position [25]. For *P. euphratica*, on the same individual, broad-ovate leaves in the upper part of the canopy have larger leaf area and leaf thickness and more developed xerophytic structures than the lanceolate leaves of the lower part [25,26]. Kenzo et al. (2015) studied 104 species in 29 families of trees in the Borneo rainforest and found that a significant linear relationship exists between leaf morphology, biochemical characteristics, and tree height [27]. Leaf structure exhibits significant differences at different tree heights, and as the tree height increases, the leaf structure exhibits more pronounced xerophytic characteristics [28]. Various studies have shown that leaf function traits are closely related to tree age and canopy height [29,30,31]. At the same time, drought has a certain impact on leaf functional traits. For morphological responses, leaf size and leaf mass area decrease in drought conditions; on the other hand, as for physiological responses, drought stress decreases the maximum photosynthesis rate and the electron transfer rate of the leaf [32]. In addition, the morphological and anatomical characteristics of leaves are closely related to physiological traits [33]; leaves with thicker Palisade cells and spongy tissue will generally show stronger photosynthetic capacities [32,33].

*Populus pruinosa* Schrenk belongs to the genus *Populus* L. of Salicaceae. It is distributed on desert riversides in the arid and semi-arid regions of the world and is an excellent tree species for wind prevention, sand fixation, and water and soil conservation. The anatomical structures of *P. pruinosa* leaves become more obvious with progressive developmental stages and increasing canopy height [26,34]. However, the relationship between the functional traits (morphology, structure, photosynthetic water physiology, osmotic substances, and endogenous hormone content) of *P. pruinosa* leaves and tree age is still unclear. Therefore, we hypothesized that there are significant differences in the functional traits of *P. pruinosa* leaves at different developmental stages and the changing trend is different with the increase in canopy height. The present study aims to provide a multifaceted measurement to understand the changes in the leaf functional traits of *P. pruinosa* in each canopy with increasing tree age and clarify the relationships between leaf functional traits and growth and development. The results provide insights into the growth of *P. pruinosa* in the desert riverside.

## 2. Results

### 2.1. Changes in the Morphological Characteristics of Leaves

**Figure 1:**
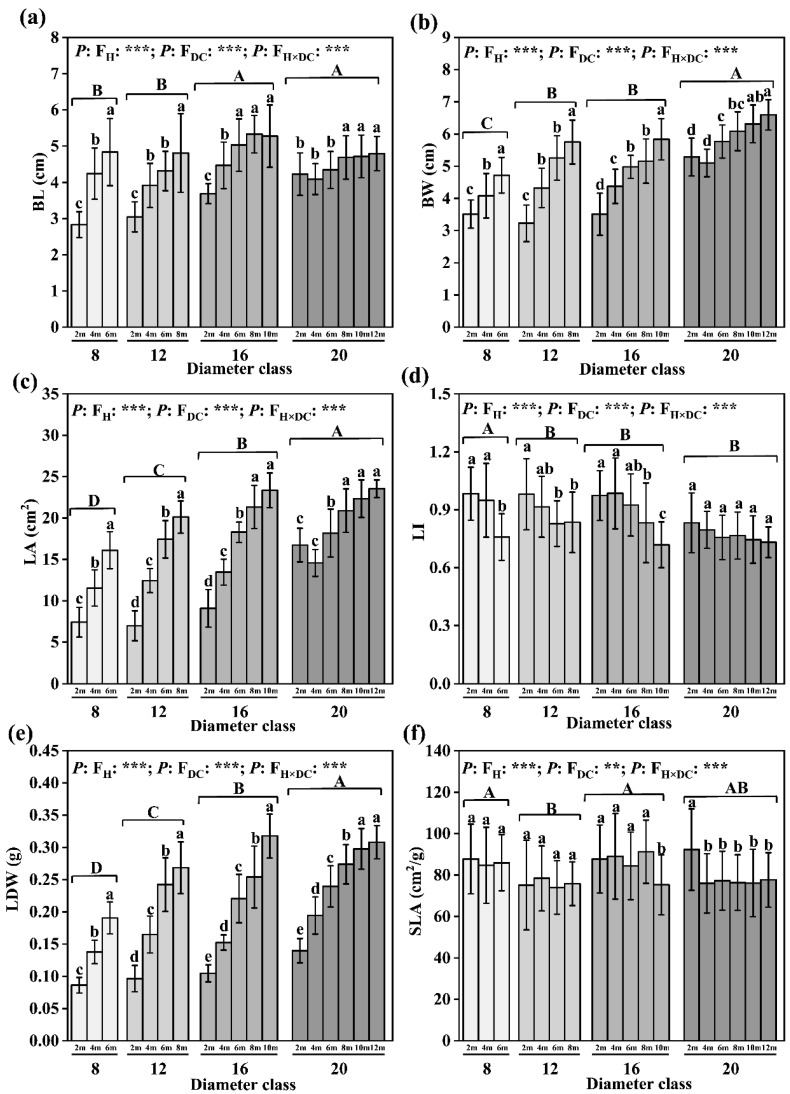
Changes in leaves’ morphological characteristics in different diameter classes of *P. pruinosa*. Note: (**a**) blade length; (**b**) blade width; (**c**) leaf area; (**d**) leaf index; (**e**) leaf dry weight; (**f**) specific leaf area. The lowercase letters and the uppercase letters represent the significance of the differences between different leaf heights and different diameter classes of *P. pruinosa* (*p* < 0.05). Two-factor analyses of variance (ANOVA) were applied to evaluate the effects of different factors and interactions. FH, leaf height effect; FDC, diameter class effect; FH×DC, leaf height × diameter class effect (*p* < 0.001: ***; 0.001 < *p* < 0.01: **).

The BL, BW, LA, and LDW increased and LI decreased with increasing diameter class, and a substantial difference was observed between classes 20 and 8 (Figure 1). From diameter classes 8 to 20, the BL, BW, LA, and LDW increased by 12.77%, 42.72%, 64.59%, and 75.19%, respectively, whereas the LI decreased by 14.16%. Among the diameter classes, the BW, LA, and LDW of class 20 were the largest, and the LI was the smallest. Class 8 had the largest SLA, which was 86.24 cm^2^/g, and class 12 had the smallest SLA, which was 12.05% smaller than the former. The BL, BW, LA, and LDW gradually increased with increasing leaf height (Figure 1).

### 2.2. Changes in Anatomical Structure Characteristics of Leaves

**Figure 2:**
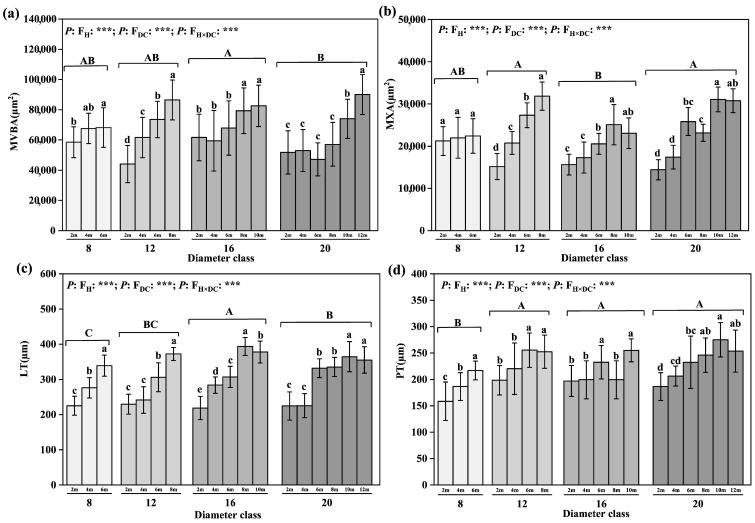
Changes in leaves’ anatomical structure characteristics in different diameter classes of *P. pruinosa*. Note: (**a**) main vein vascular bundle area; (**b**) main vein xylem area; (**c**) leaf thickness; (**d**) palisade tissue thickness. The lowercase letters and the uppercase letters represent the significance of the differences between different leaf heights and different diameter classes of *P. pruinosa* (*p* < 0.05). Two-factor analyses of variance (ANOVA) were applied to evaluate the effects of different factors and interactions. FH, leaf height effect; FDC, diameter class effect; FH×DC, leaf height × diameter class effect (*p* < 0.001: ***).

After a comparison of the anatomical structure characteristics of each diameter class (Figure 2), we found that the LT and PT in classes 8 to 20 increased by 5.05% and 21.79%, respectively, and the difference was significant. The VBA, LT, PT, and MXA of the other diameter classes, except class 8, of leaves increased with increasing leaf height and at the highest leaf height (Figure 2). A significant difference existed between the layer and the 2 m leaf height. From the lowest leaf height to the highest leaf height, the largest increases in the MVBA and the MXA appeared in class 20, of 73.99% and 113.5%, respectively, while the largest increase in the LT, of 72.74%, appeared in class 16. The PT increased by 36.77%, 27.29%, 29.49%, and 36.12%, respectively, from class 8 to class 20. The MVBA and the LT appeared in class 16, with values of 70,175.42 and 323.33 µm, respectively; class 20 had the largest MXA, of 23,607.83 µm, which was significant and considerably larger than that of class 8.

### 2.3. Changes in Photosynthetic Physiological Parameters of Leaves

**Figure 3:**
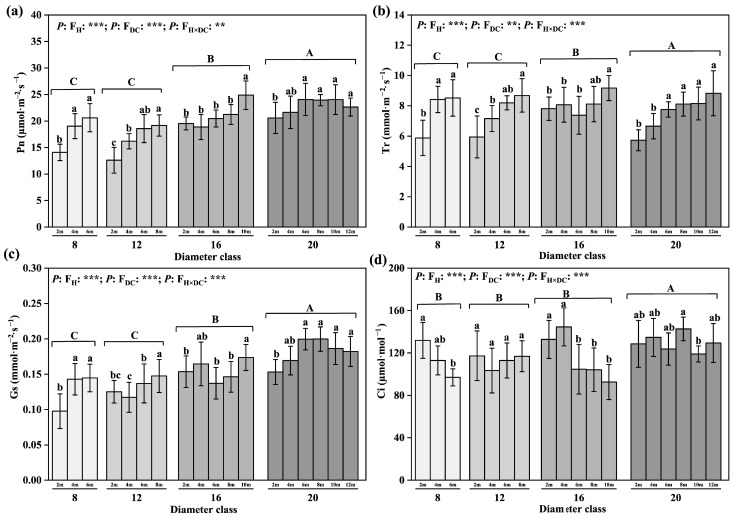
Changes in leaves’ photosynthetic physiological parameters in different diameter classes of *P. pruinosa*. Note: (**a**) photosynthetic rate; (**b**) transpiration rate; (**c**) stomatal conductance; (**d**) intercellular CO_2_ concentration. The lowercase letters and the uppercase letters represent the significance of the differences between different leaf heights and different diameter classes of *P. pruinosa* (*p* < 0.05). Two-factor analyses of variance (ANOVA) were applied to evaluate the effects of different factors and interactions. FH, leaf height effect; FDC, diameter class effect; FH×DC, leaf height × diameter class effect (*p* < 0.001: ***; 0.001 < *p* < 0.01: **).

Through comparison of light and abilities of leaves from different diameter classes (Figure 3), we found that the Pn and the Gs increased by 37.54% and 41.5% from class 8 to 20, respectively, and the difference was significant. No significant difference in Tr was detected among the diameter classes, but the Ci of class 20 was significantly higher than those of classes 8, 12, and 16. The Pn, Tr, and Gs values of the leaves of each diameter class gradually increased with increasing leaf height; the difference between the highest leaf height of each diameter class and the 2 m leaf height was significant, whereas the Ci showed a decreasing trend (Figure 3). The difference between the highest leaf height of class 16 and the 2 m leaf height was significant. For example, in diameter class 20, the Pn, Tr, and Gs increased by 9.94%, 54.25%, and 18.97%, respectively, from 2 m to 12 m. The largest increase in Gs, of 48.18%, occurred in class 8. The Ci decreased the most in class 16, by 30%.

### 2.4. Changes in Water Use Efficiency of Leaves

**Figure 4:**
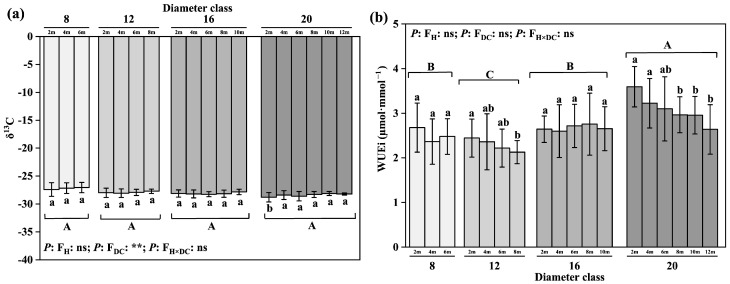
Changes in leaves’ water use efficiency in different diameter classes of *P. pruinosa*. Note: (**a**) stable carbon isotope value; (**b**) instantaneous water use efficiency. The lowercase letters and the uppercase letters represent the significance of the differences between different leaf heights and different diameter classes of *P. pruinosa* (*p* < 0.05). Two-factor analyses of variance (ANOVA) were applied to evaluate the effects of different factors and interactions. FH, leaf height effect; FDC, diameter class effect; FH×DC, leaf height × diameter class effect (*p* < 0.01: **; *p* > 0.05: ns).

By comparing the water use efficiencies of different diameter classes (Figure 3), we found no significant difference in the δ^13^C value among the diameter classes, whereas the WUEi increased with the diameter class, to 36.56% higher than that of the former. The δ^13^C of the leaves increased with the leaf height in class 20. From 2 m to 12 m, the δ^13^C increased by 2.01%; the difference between the highest leaf height and the 2 m leaf height was significant (Figure 4). The increase in the WUEi with the leaf height showed a decreasing trend from 2 m to 12 m, with a corresponding decrease of 26.6%. The difference between the highest leaf height and the 2 m leaf height was significant.

### 2.5. Changes in Physiological Characteristics of Leaves

**Figure 5:**
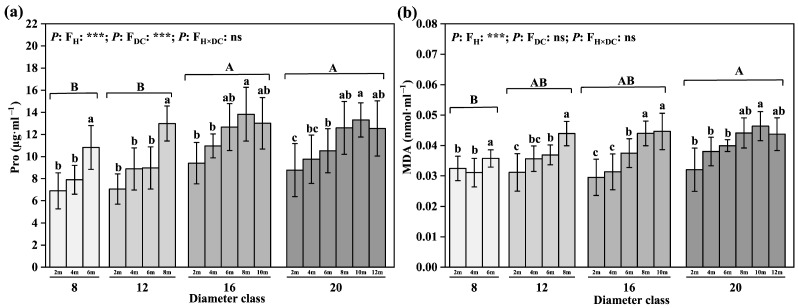
Changes in leaves’ physiological characteristics in different diameter classes of *P. pruinosa*. Note: (**a**) proline content; (**b**) malondialdehyde content. The lowercase letters and the uppercase letters represent the significance of the differences between different leaf heights and different diameter classes of *P. pruinosa* (*p* < 0.05). Two-factor analyses of variance (ANOVA) were applied to evaluate the effects of different factors and interactions. FH, leaf height effect; FDC, diameter class effect; FH×DC, leaf height × diameter class effect (*p* < 0.001: ***; *p* > 0.05: ns).

The comparison of the proline and malondialdehyde contents in leaves of different diameter classes (Figure 5) shows that the contents of Pro and MDA increased by 31.71% and 22.93% from class 8 to class 20, respectively. The difference in class 8 was significant; the Pro values of classes 16 and 20 were significantly higher than those of classes 8 and 12. The MDA contents of classes 12, 16, and 20 was significantly higher than that of class 8. In each diameter class, the leaf Pro and MDA contents increased with increasing leaf height, and the difference between the highest leaf height and the 2 m leaf height in each diameter class was significant (Figure 5). From 2 m to the highest leaf height, the largest increase in Pro appeared in class 12, with a value of 83.88%, while the greatest increase in the MDA content appeared in class 16, with a value of 51.03%.

### 2.6. Changes in Endogenous Hormones Content of Leaves

**Figure 6:**
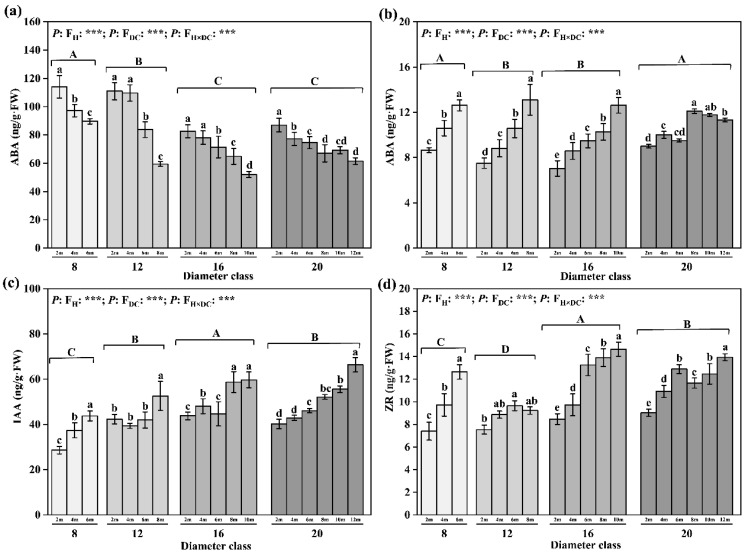
Changes in leaves’ endogenous hormone content in different diameter classes of *P. pruinosa*. Note: (**a**) abscisic acid content; (**b**) gibberellin content; (**c**) indoleacetic acid content; (**d**) zeatin riboside content. The lowercase letters and the uppercase letters represent the significance of the differences between different leaf heights and different diameter classes of *P. pruinosa* (*p* < 0.05). Two-factor analyses of variance (ANOVA) were applied to evaluate the effects of different factors and interactions. FH, leaf height effect; FDC, diameter class effect; FH×DC, leaf height × diameter class effect (*p* < 0.001: ***).

After comparing the contents of endogenous hormones in leaves of different diameter classes (Figure 6), we found that the contents of IAA and ZR showed an increasing trend with increasing diameter class; meanwhile, the content of ABA showed a decreasing trend. From class 8 to class 20, the ABA content decreased by 27.45%, while the contents of IAA and ZR increased by 15.31% and 15.81%, respectively, and the maximum values of IAA and ZR appeared in class 16, with values of 51.95 and 12.95 ng/g·FW, respectively, and were considerably larger than the rest. In each diameter class, the contents of endogenous hormones GA_3_, IAA, and ZR in heteromorphic leaves increased with increasing leaf height; by contrast, the ABA content decreased with increasing leaf height, and the differences among layers were significant (Figure 6). With increasing leaf height, the maximum decrease in the ABA content appeared in class 12 (46.48%), whereas the maximum increases in the GA_3_ and ZR contents appeared in class 16 (79.28% and 72.87%, respectively). From class 8 to class 20, from the lowest leaf height to the highest leaf height, the IAA content increased by 53.01%, 24.27%, 36.4%, and 65.21%, respectively.

### 2.7. Relationship between the Functional Characteristics of Leaves

**Figure 7:**
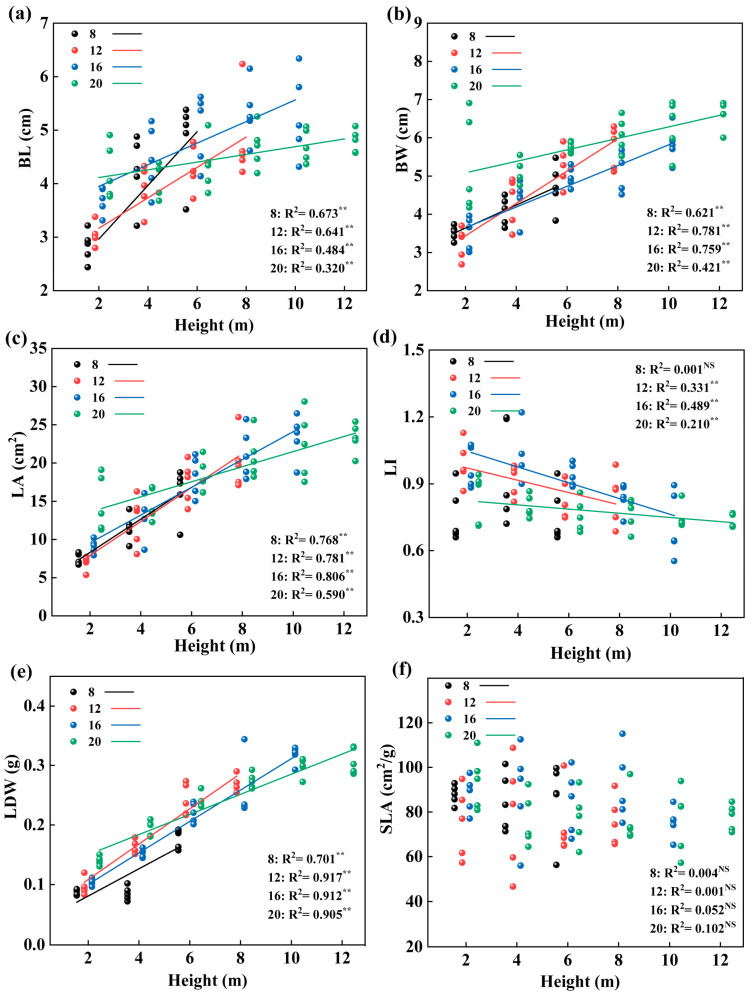
Linear fitting of leaf morphology characteristics of different diameter classes (8/12/16/20) with leaf height of *P. pruinosa*. (*p* < 0.01: **; *p* > 0.05: NS). Note: (**a**) BL, blade length; (**b**) BW, blade width; (**c**) LA, leaf area; (**d**) LI, leaf index; (**e**) LDW, leaf dry weight; (**f**) SLA, specific leaf area.

**Figure 8:**
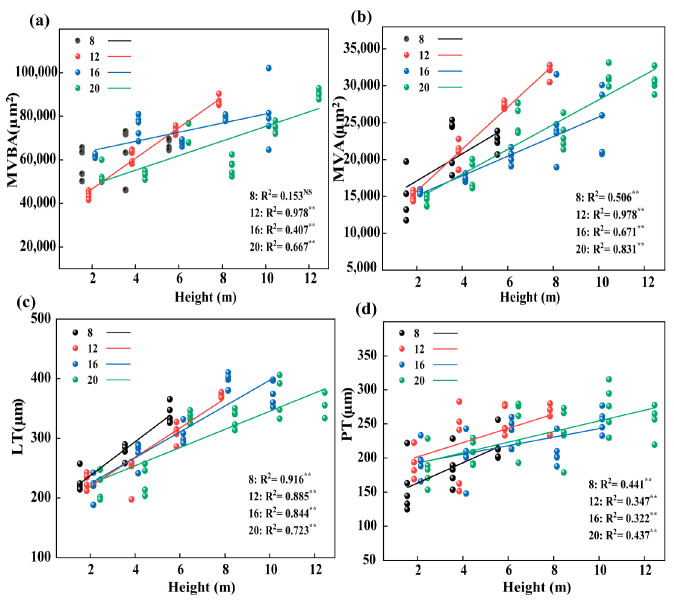
Linear fitting of leaf anatomical structure characteristics of different diameter classes (8/12/16/20) with leaf height of *P. pruinosa*. (*p* < 0.01: **; *p* > 0.05: NS) Note: (**a**) MVBA, main vein vascular bundle area; (**b**) MXA, main vein xylem area; (**c**) LT, leaf thickness; (**d**) PT, palisade tissue thickness.

**Figure 9:**
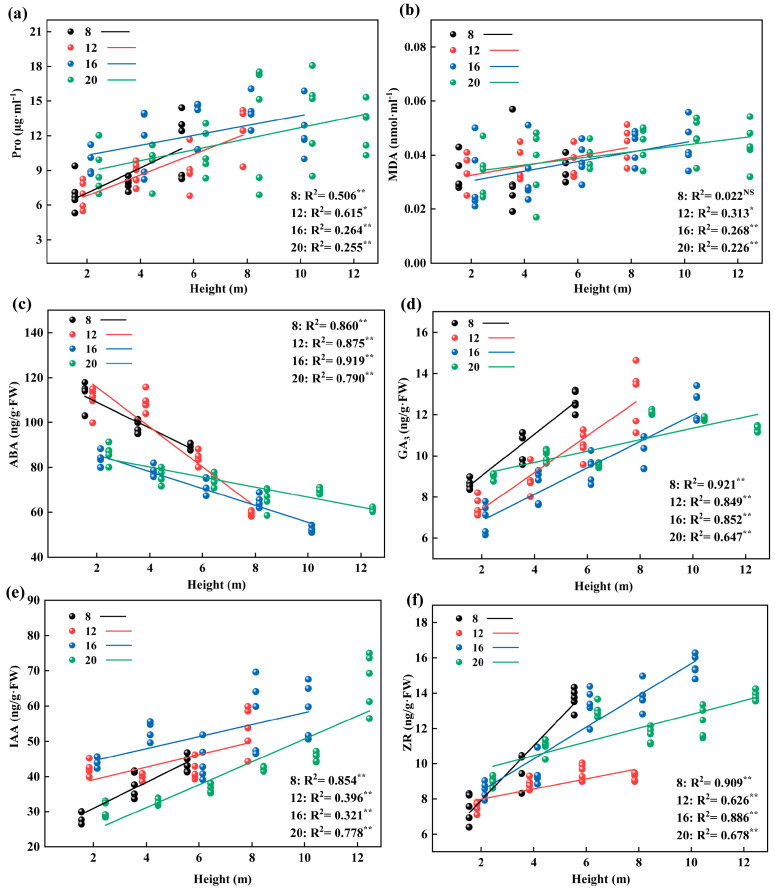
Linear fitting of leaf physiological and biochemical characteristics of different diameter classes (8/12/16/20) with leaf height of *P. pruinosa*. (*p* < 0.01: **; 0.01 < *p* < 0.05: *; *p* > 0.05: NS) Note: (**a**) Pro, proline; (**b**) MDA, malondialdehyde; (**c**) ABA, abscisic acid; (**d**) GA_3_, gibberellin; (**e**) IAA, indoleacetic acid; (**f**) ZR, zeatin riboside.

According to the linear fitting of leaf traits of different diameter classes with tree height, we found that the BL, BW, LA, LDW, MVA, LT, PT, Pro, GA_3_, IAA, and ZR values of the four diameter classes increased linearly with increasing tree height (*p* < 0.05, Figure 7a–c,e, Figure 8b–d, and Figure 9a,d–f). The ABA decreased linearly with tree height (*p* <0.05, Figure 9c). The difference is that the leaf traits of LI, MVBA, and MDA only had no significant relationship to tree height in the 8th diameter class (Figure 7d,e and Figure 8b). At the same time, we found that the change rate of most leaf traits in diameter classes 8 and 12 was faster with increasing tree height. The BW, LA, LDW, MVBA, MVA, and MDA changed fastest in diameter class 12 (*p* < 0.05, Figure 7b,c,e, Figure 8a,b, and Figure 9b), while the LT, the PT, and hormone characteristics GA_3_, IAA, and ZR changed the fastest in diameter class 8 (*p* < 0.05, Figure 8c,d and Figure 9d–f).

Based on correlation analysis according to different diameter classes (Figure S1), the BL, BW, LA, LDW, LT, GA_3_, IAA, and ZR in the four diameter orders were significantly positively correlated with tree height; the ABA was significantly negatively correlated with tree height. The difference is the lack of significant correlation between the MVBA, the MXA, and tree height in diameter class 8, while a significant positive correlation exists between the MVBA and MXA values with tree height in diameter classes 12, 16, and 20.

**Figure 10:**
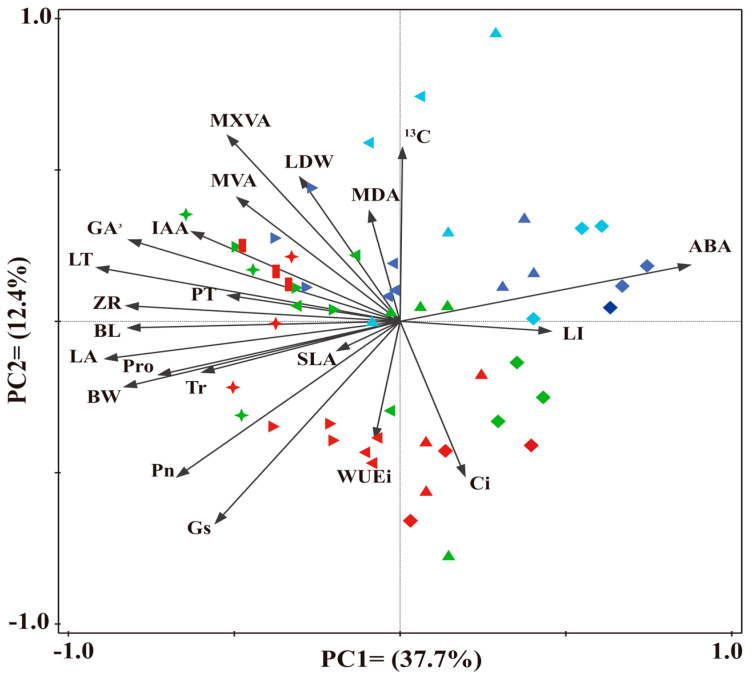
Principal component analysis (PCA) based on leaf traits in *P. pruinosa* at different heights across four diameter classes. The diamond, upper triangle, right triangle, and left triangle represent the tree heights of 2, 4, 6, 8, 10, and 12 m, respectively; the light blue, dark blue, green, and red represent diameter classes 8, 12, 16, and 20, respectively. BL, blade length; BW, blade width; LA, leaf area; LI, leaf index; LDW, leaf dry weight; SLA, specific leaf area; MVBA, main vein vascular bundle area; MXA, main vein xylem area; LT, leaf thickness; PT, palisade tissue thickness; Pn, photosynthetic rate; Tr, transpiration rate; Gs, stomatal conductance; Ci, intercellular CO_2_ concentration; δ^13^C, stable carbon isotope value; WUEi, instantaneous water use efficiency; Pro, proline; MDA, malondialdehyde; ABA, abscisic acid; GA_3_, gibberellin; IAA, indoleacetic acid; ZR, zeatin riboside.

Overall, the BL, BW, LA, LDW, Pn, Gs, Ci, WUEi, and Pro correlated significantly with diameter class, and the contents of δ^13^C and ABA had significant negative correlations (Figure S2); the BL, BW, LA, LDW, MVBA, MXA, LT, PT, Pn, Tr, Gs, Pro, MDA, GA_3_, IAA, and ZR had significant correlations with leaf height, whereas the LI and ABA contents correlated significantly with leaf height. In addition, the contents of GA_3_, IAA, and ZR correlated significantly positively with the contents of Pro and MDA, whereas the content of ABA correlated significantly negatively with the latter and leaf height and diameter class had significant interactive effects on it. The results show that heteromorphic leaf functional traits are closely related to developmental stages, the canopy heights of leaves, and the relationship between the two. The first two axes of the PCA explain 50.1% of the total variance of the studied leaf traits in four *P. pruinosa* diameter classes (Figure 10). In these four diameter classes of leaves, the PC1 was strongly influenced by the BL, LA, LT, PT, ABA, and ZR, while the PC2 was strongly influenced by the δ^13^C, WUE_i_, LDW, C_i_, and MDA. In addition, Figure 10 shows the high contents of ABA and LI in diameter classes 8 and 12, respectively, with high Pn, Gs, PT, LT, LA, IAA, and ZR values in diameter classes 16 and 20.

## 3. Discussion

### 3.1. Differences in and Relationships of Leaves’ Functional Traits at Different Developmental Stages

Differences in the special adaptive structures and functions of leaves may be caused in the same species under different ecological conditions or related to the crown position and the growth stage [19,24,28,35]. Differences in the morphologies and physiological characteristics of the scale and needle leaves of *Sabina vulgaris* are related to the growth stage. At the seedling stage, they grow under the canopy and only have needle leaves, whereas mature individuals grow in an open environment with needle and scale leaves. Compared with needles, scale leaves have a larger LMA, a higher leaf area photosynthetic rate, higher water use efficiency, and stronger resistance to photoinhibition [24]. Another typical case related to differences in leaf morphologies and physiological characteristics related to the crown position and the growth stage is the study of the morphologies and physiological characteristics of the heteromorphic leaves of *P. euphratica*. Significant differences existed in the morphological structures and physiological functions and are related to the ontogeny stage and the coronal position [25]. This finding revealed the differences in the types and functions of the heteromorphic leaves of *P. euphratica* at different developmental stages. The morphological and anatomical structures of the heteromorphic leaves of *P. euphratica*, such as the LA, LT, LDW, and PT, increased with increasing diameter class, and all correlated significantly with the diameters, at breast height, at different developmental stages. With age, the factor of heterogeneity of microenvironments and the factor of competition were more strongly pronounced [14]. The results of this study were similar to those for *P. euphratica*. The differences in the morphologies, structures, and physiological functions of heteromorphic leaves of *P. pruinosa* are related to developmental stages. The LA, Pn, Gs, WUEi, and Pro correlated significantly with diameter class, and the contents of δ^13^C and ABA correlated significantly negatively (Figure S2). Leaf area expansion is closely related to some major plant physiological events [36], such as photosynthesis, transpiration, and carbon flow, which are considered important requirements to enlarge light harvesting [37]. In the leaves of low-diameter *P. pruinosa*, where LA is small, as a *P. pruinosa* individual grows, more energy is needed to sustain nutritional growth and also to allocate energy to reproductive organs, such as flowers and seeds, which require individual trees to increase their productivity. With the large-diameter class of *P. pruinosa*, the LA was relatively large, the photosynthetic ability was strong, and the changes in the contents of Pro, MDA, and endogenous hormones also provided infiltration conditions for the water supply.

### 3.2. Differences in Functional Traits of Leaves at Different Canopy Heights and the Relationship to Canopy Height

Leaves represent highly plastic structures that respond to environmental gradients existing in plant canopies [38,39]. Differences in special adaptive structures and functions of leaves in woody plants are not only related to the growth stage but also to the coronal part [19,28]. Leaf morphology and physiological and biochemical characteristics vary with tree height, owing to changes in light availability and evapotranspiration requirements during ontogeny [40]. Leaf morphological structure exhibits more apparent xerophytic characteristics with increasing canopy height [25,28]. For example, to cope with the water stress caused by tree height, the leaf area of *Parashorea chinensis* Wang Hsie decreases with increasing tree height, and the palisade structure increases with increasing tree height. Such increases exhibit stronger xerophyte structures [28]. The proportions of scales and needles of *Sabina vulgaris* in the canopy of mature individuals changed significantly with a change in canopy position, and the scales at the top of the canopy had a higher photosynthetic rate, a stronger photosystem I capacity, and higher carbon in the light-saturated state, as well as a higher isotope ratio (δ^13^ C) and a higher water use efficiency than the needles [24]. Studies on *Eucalyptus amygdalina* F. Muell. found that the area of transpiration and photosynthesis decreased with increasing tree age and height in response to water stress [19]. Narayan’s research on apple quality showed that the net photosynthetic rate of the low canopy was lower than that of the high canopy, but after treatment with summer pruning and installation of reflective film, the net photosynthetic rate of the low canopy was significantly increased due to the increased amount of light, thereby improving the fruit quality [41]. The morphology, anatomical structure, and physiological characteristics of the heteromorphic leaves of *Populus euphratica* Olivier are related to the ontogeny stage and the coronal position. With increasing canopy height, the leaf area, leaf thickness, specific leaf weight, palisade tissue thickness, net photosynthetic rate, transpiration rate, stomatal conductance, and proline and malondialdehyde contents of the sampled height increased and correlated significantly positively with that height [25]. The LA, LDW, MVBA, LT, PT, net Pn, Tr, Gs, ABA, and MDA and endogenous hormones GA_3_, IAA, and ZR increased with increasing leaf height and correlated significantly positively with it. Within forest canopies, environmental gradients exist and enhance water losses in the direction of the treetops [42]. A range of anatomical traits (xylem cross-sectional area, number and dimensions of xylem conduits, leaf thickness, etc.) modulate the efficiency of water transfer from the stem xylem through the petiole, the veins, and the extravascular paths to the guard and mesophyll cells [43,44,45,46]. Increases in leaf area, leaf thickness, and palisade cells increase the photosynthetic area and the thickness of the palisade cell as the leaf height increases, leading to the enhancement of the photosynthetic capacity [33]. δ^13^C is the proxy of WUE and plant water relations and a useful method to determine drought resistance to provide insights into the chemical, physical, and metabolic processes involved in carbon transformation in stressed plants, which determines the water utility and water conservation status for the forest seedlings [32]. Narayan’s study on *Prunus sargentii* Rehder and *Larix kaempferi* Carr. seedlings under drought stress discovered that δ^13^C showed a decreasing trend [32], but our study found that in the leaves of *P. pruinosa*, there was no significant difference in δ^13^C among different canopies and developmental stages, possibly because the environmental conditions were not enough to cause significant changes in δ^13^C. As the leaf height increased, the productivity and water transport efficiency of *P. pruinosa* increased, as well as the abilities to obtain and use nutrients and fix carbon; as such, more photosynthetic products were used to build defense tissues and accumulate dry matter. Changes in the anatomical structure and osmoregulatory substances enhanced water storage in the body, maintained water balance, and enhanced defense against environmental stresses by regulating the leaf area, growth rate, and nutrient storage; reducing water loss; and enhancing drought tolerance.

Tree growth results in longer vertical water-conducting paths from the roots to the canopy, requiring a greater pulling force and greater moisture gradients [47]. In higher plants, water stress induces accumulation of osmotic regulators such as proline (Pro) and malondialdehyde content (MDA) [48]. Hormones can also induce accumulation of soluble osmotic substances by regulating the intracellular metabolism and enhancing the survival of plants under stress [49,50]. Our results showed that with increasing leaf height of *P. pruinosa* of different developmental stages, the contents of the osmotic regulator proline and the growth hormones GA_3_, IAA, and ZR increased significantly, while the ABA content decreased significantly. With increased tree height, the leaves at the top of the canopy need a stronger osmotic regulation ability to enhance the power of water absorption. An increase in endogenous hormone content can induce accumulation of osmotic regulatory substances. This synergistic effect provides a strong osmoregulation ability. When plants are under stress, an increase in ABA content reduces leaf stomatal conductance, transpiration water loss, and CO_2_ absorption and fixation, which has a certain inhibitory effect on plant growth [51]. In contrast to our results, the ABA contents of *P. pruinosa* of different diameter classes decreased significantly with increasing tree height. This decrease may be caused by an increase in the transpiration rate, and the synergistic changes of endogenous hormones and osmoregulatory substances play an important role in maintaining water balance.

## 4. Materials and Methods

### 4.1. Study Area

The study area, located in the northwestern margin of the Tarim Basin in Xinjiang Province of China (81°17′56.52″ E, 40°32′36.90″ N, 980 m a.s.l.), has a typical temperate desert climate. The average annual rainfall is approximately 50 mm, the potential evaporation reaches up to 1900 mm, the yearly average temperature is 10.8 °C, and the average annual sunshine duration is 2900 h. The *P. euphratica* and *P. pruinosa* mixed forest at the study site covers an area of 180.6 ha, with a groundwater level of 1.5 m.

### 4.2. Experimental Design and Sampling

The diameter at breast height is 4 cm, which is the class, and the four diameter classes of 8 (5.9–9.9 cm), 12 (10–13.9 cm), 16 (14–17.9 cm), and 20 (18–21.9 cm) represent different developmental stages (tree age). Five sample trees with uniform crowns were selected from each diameter class, with a total of 20 trees (Table 1). The diameter class represents the developmental stage, and the leaf height represents the height of the canopy where leaves are located. Leaf samples were collected from the *Populus pruinosa* forest of Tarim University and stored in the Key Laboratory of Tarim University (voucher numbers: 2019-*Populus pruinosa*- [8/12/16/20]) (Table S1).

Sampling points were selected from the trunk bases of the sampled trees along the tree height (H) at 2 m intervals. The points were distributed at 2, 4, 6, 8, 10, and 12 m from the sample trees, as well as the height of the heteromorphic leaves in the vertical space of the canopy. At each sampling point, 1-year-old branches were collected from four directions (east, south, west, and north). A total of 30 branches were collected at each point, and leaves were collected at the fourth nodes from the bases of the branches to analyze the morphology, anatomy, dry mass, δ^13^C, and concentrations of proline (Pro) and malondialdehyde (MDA). The leaves used for the analyses of Pro, MDA, and the endogenous hormones were stored in liquid nitrogen after collection.

### 4.3. Measurements of Leaf Morphological and Anatomical Parameters

There were 18 sampling sites in 4 developmental stages. A total of 30 branches were collected at each point, and leaves were collected at the fourth nodes from the bases of the branches to analyze morphology. We used the blade length-to-width ratio (leaf index) to assess changes in leaf shape. Blade length (BL), blade width (BW), and leaf area were measured using using a SCANNER (MRS-9600TFU2, Shanghai, China) and LA-S plant image analysis software. The leaf index was calculated using the blade length/blade width ratio.

A total of 4 branches were collected at each point, and leaves were collected at the fourth nodes from the bases of the branches to analyze anatomical parameters (n = 360). The blade was cut transversely at its widest point. The material that retained the primary vein and leaf margin was fixed in a formalin–acetic acid–alcohol (FAA) solution. Tissue sections were prepared as 8 µm-thick paraffin sections, double-stained with saranine–fast green, and mounted in a neutral resin. The main vein vascular bundle area, main vein xylem area, leaf thickness, and palisade tissue thickness were measured using a Leica microscope (Leica DM4 B, Wetzlar, Germany). Five fields of view were observed for each leaf, and 20 values were obtained for each field of view. The average values of the leaf structural parameters in the five fields of view were collected as anatomical parameters [25].

### 4.4. Measurement of Leaf Dry Mass

After leaf morphology treatment, the samples were placed in a preheated oven. The oven temperature was increased to 105 °C to deactivate the samples for 10 min. The oven temperature was then lowered to 65 °C, at which the samples were dried to a constant weight. Paper bags containing the material were removed and placed in a desiccator. After cooling to room temperature, the samples were weighed using an electronic balance (0.001 g). Leaf mass per area (LMA) was calculated based on leaf area and dry mass.

### 4.5. Measurement of Leaf Photosynthesis

A total of 4 branches were collected at each point, and leaves were collected at the fourth nodes from the bases of the branches to analyze photosynthesis parameters (n = 360). One-year-old branches were collected with pruning shears and immediately wrapped in plastic wrap to cover the incisions. Photosynthetic gas exchange characteristics were measured with a portable photosynthesis system, LI-COR 6400XT (LI-COR, Lincoln, NE, USA), for each fourth fully expanded leaf between 09:00 a.m. and 12:00 a.m. on 20 and 28 July 2019. The light-saturated net photosynthesis rate (Pn), stomatal conductance (Gs), intercellular CO_2_ concentration (Ci), and transpiration rate (Tr) were measured under the following conditions: leaf temperature, 25 °C; relative air humidity, 60%; ambient CO_2_ concentration, 400 ± 5 μmol CO_2_ mol^−1^; and photosynthetic photon flux density, 1250 μmol m^−2^ s^−1^. The instantaneous water use efficiency (WUEi) of the leaves was calculated as WUEi = Pn/Tr. Ten leaves from each sampling point were measured in triplicate [25].

### 4.6. Measurement of Stable δ^13^C

The leaves collected at each sampling point were kept separately in paper bags and placed in a preheated oven; the samples were immediately rinsed with distilled water and deactivated in an oven at 105 °C (n = 270). The samples were oven-dried at 60 °C for 48 h to a constant weight. The dried samples were pulverized using a pulverizer and passed through a 90-mesh sieve. The carbon isotope composition of the purified gas was analyzed using a stable gas isotope mass spectrometer (Thermo Fisher Scientific, Inc., Waltham, MA, USA). Additional details are described by Zhai et al. [25].

### 4.7. Measurement of MDA and Pro Concentrations

The leaves (without petioles) at the fourth nodes of the branches were collected, quickly frozen with liquid nitrogen and stored at −80 °C (n = 270). The acid ninhydrin method was used to determine the leaf proline content (µg/g). The MDA content (µmol/g) was determined with the thiobarbituric acid color method. Additional details are described by Zhai et al. [25].

### 4.8. Measurement of Hormone Content

An enzyme-linked immunoassay was used to determine the contents of abscisic acid (ABA), gibberellin (GA_3_), indoleacetic acid (IAA), and zeatin riboside (ZR) at China Agricultural University.

### 4.9. Statistical Analysis

Statistical analyses were performed using the Statistical Package for the Social Sciences (SPSS, Chicago, IL, USA) version 18.0. All data were checked for normality and homogeneity of variances and log-transformed to correct deviations from these assumptions when necessary. Tukey HSD tests were conducted to detect significant differences among the treatments. Two-factor analysis of variance (ANOVA) was performed to analyze the effects of canopy height, DBH, and their interactions. All differences were considered statistically significant at *p* < 0.05.

## 5. Conclusions

The morphological structures and physiological characteristics of the heteromorphic leaves of *P. pruinosa* had evident canopy height gradient differences in each diameter class, and the leaf morphological structures and physiological characteristics showed more apparent xerophytic structure characteristics with advancing developmental stages and increasing canopy height gradients. *P. pruinosa* heteromorphic leaves are used to maintain material and energy requirements by regulating each functional trait, improving resource utilization efficiency, and enhancing defense against environmental stresses. This study was designed at different developmental stages to comprehensively investigate the changes in the functional traits of *P. pruinosa* leaves, including leaf morphology, anatomical structure, and physiological characteristics, providing theoretical support for desertification control.

## Figures and Tables

**Table 1:** Sample-tree-related information.

	Information	Diameter Class	Average Diameter at Breast Height (cm)	Average Height of First Branch (cm)	Average Tree Height (m)
Sample Tree Number					
8–3		8	7.51	152.51	7.42
12–5		12	12.13	155.73	9.15
16–4		16	16.07	177.13	10.83
20–7		20	19.6	188.57	12.90

## Data Availability

The datasets generated for this study are available on request to the corresponding author.

## Supplementary Materials

The following supporting information can be downloaded at: https://www.mdpi.com/article/10.3390/plants12122262/s1, Table S1: Sample tree number related information; Figure S1: Correlation analysis according to different diameter classes; Figure S2: Correlation analysis of δ^13^C and ABA content.
