# Supplementary material for: Differences in the Functional Traits of Populus pruinosa Leaves in Different Developmental Stages
Source: Plants (Basel). 2023 Jun 9;12(12):2262. doi: 10.3390/plants12122262 (PMC10304746; doi:10.3390/plants12122262)
Supplement: Supplementary file 1 [file plants-12-02262-s001.zip › Supplementary Table S1.pdf]

Supplementary Table S1 Sample tree number related information

Information	Sample	Diameter class	Voucher numbers
2019-*Populus pruinosa*-[8/12/16/20]	8-3	8	8-3(1)
			8-3(2)
			8-3(3)
			8-3(4)
			8-3(5)
	12-5	12	12-5(1)
			12-5 (2)
			12-5 (3)
			12-5 (4)
			12-5 (5)
	16-4	16	16-4(1)
			16-4 (2)
			16-4 (3)
			16-4 (4)
			16-4 (5)
	20-7	20	20-7 (1)
			20-7 (2)
			20-7 (3)
			20-7 (4)
			20-7 (5)
