# Supplementary figures and images for: Differences in the Functional Traits of Populus pruinosa Leaves in Different Developmental Stages
Source: Plants (Basel). 2023 Jun 9;12(12):2262. doi: 10.3390/plants12122262 (PMC10304746; doi:10.3390/plants12122262)

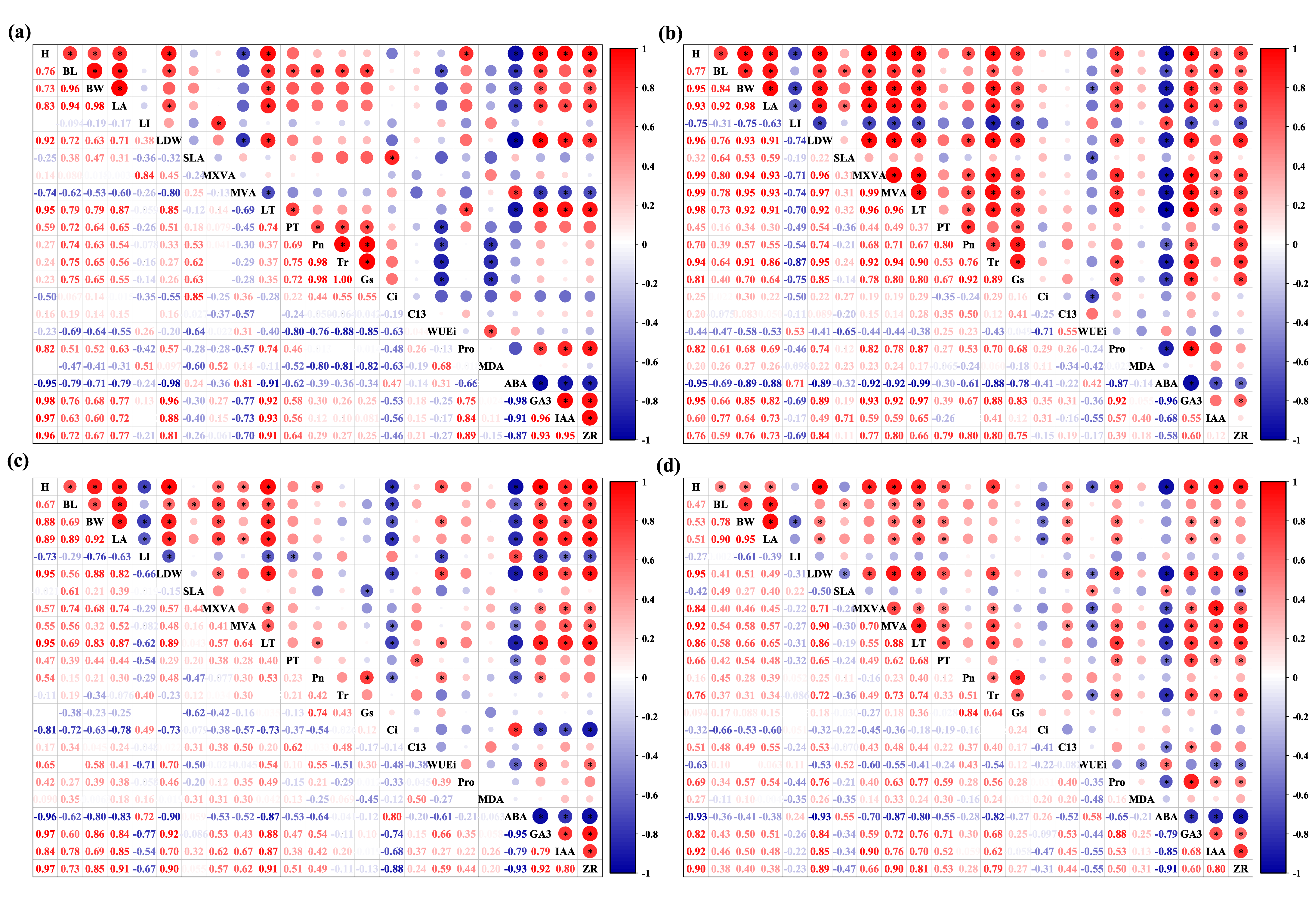

Supplement: Supplementary file 1 [file plants-12-02262-s001.zip › Fig. S1.tif]

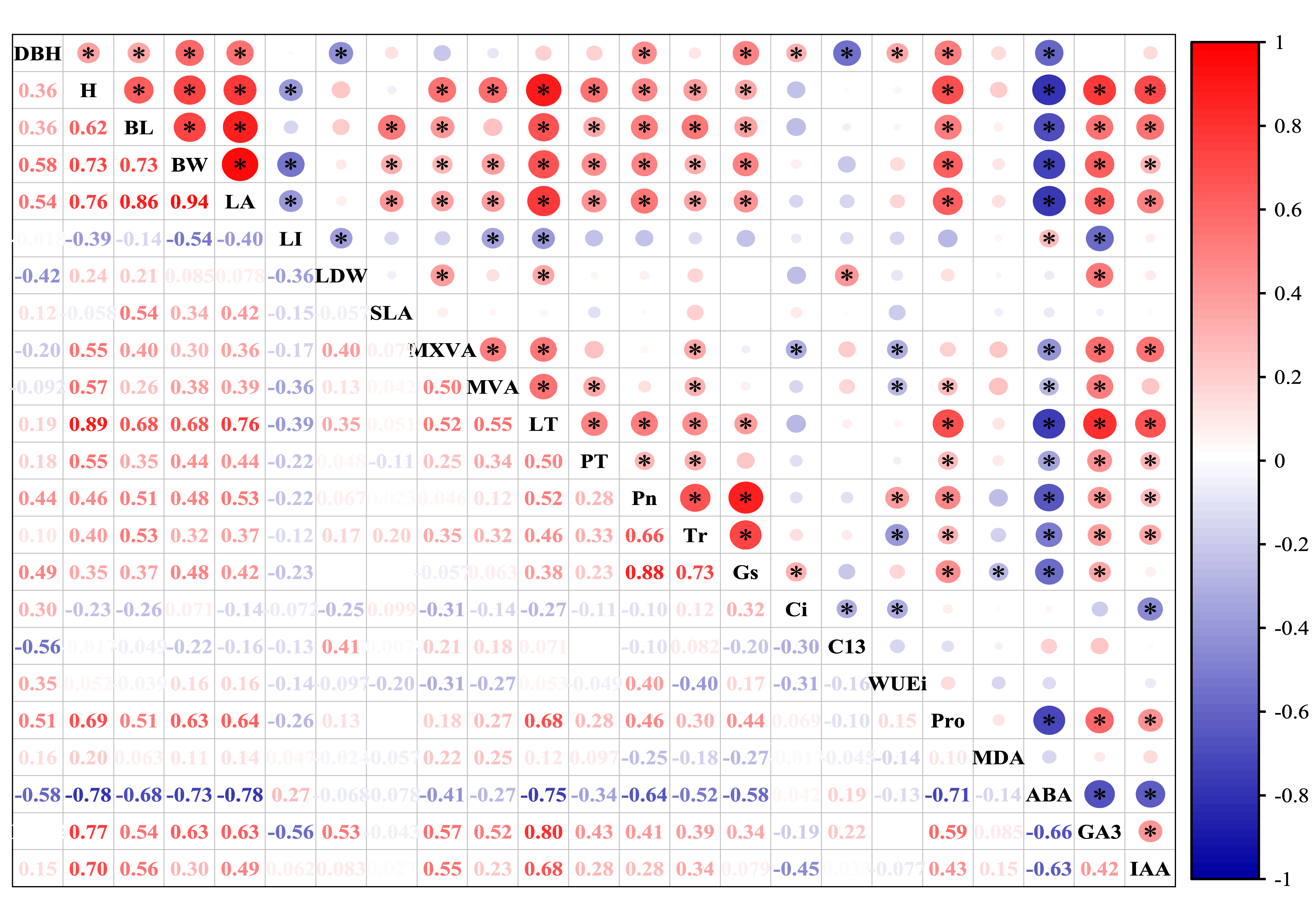

Supplement: Supplementary file 1 [file plants-12-02262-s001.zip › Fig. S2.tif]
